# Multisession Cognitive Bias Modification Targeting Multiple Biases in Adolescents with Elevated Social Anxiety

**DOI:** 10.1007/s10608-018-9912-y

**Published:** 2018-04-27

**Authors:** Stephen C. Lisk, Victoria Pile, Simone P. W. Haller, Veena Kumari, Jennifer Y. F. Lau

**Affiliations:** 1 Department of Psychology, Institute of Psychiatry, Psychology and Neuroscience, King’s College London, London, SE5 8AF UK; 2 Department of Experimental Psychology, University of Oxford, Oxford, UK; 3 Centre for Cognitive Neuroscience, College of Health and Life Sciences, Brunel University London, London, UK

**Keywords:** Social anxiety, Adolescence, CBM training, Attention bias, Interpretation bias

## Abstract

Research studies applying cognitive bias modification of attention (CBM-A) and interpretations (CBM-I) training to reduce adolescent anxiety by targeting associated cognitive biases have found mixed results. This study presents a new multi-session, combined bias CBM package, which uses a mix of training techniques and stimuli to enhance user-engagement. We present preliminary data on its viability, acceptability and effectiveness on reducing symptoms and biases using an A–B case series design. 19 adolescents with elevated social anxiety reported on their social anxiety, real-life social behaviours, general anxiety, depression, and cognitive biases at pre/post time-points during a 2-week baseline phase and a 2-week intervention phase. Retention rate was high. Adolescents also reported finding the CBM training helpful, particularly CBM-I. Greater reductions in social anxiety, negative social behaviour, and general anxiety and depression, characterised the intervention but not baseline phase. There was a significant correlation between interpretation bias change and social anxiety symptom change. Our enhanced multi-session CBM programme delivered in a school-setting appeared viable and acceptable. Training-associated improvements in social anxiety will require further verification in a study with an active control condition/group.

## Introduction

Social anxiety is prevalent in youth (Wittchen et al. 1999), can disrupt academic performance and interpersonal interactions (Owens et al. 2008), persist into adulthood, and impact other disabling mental health conditions and quality of life (Woodward and Fergusson 2001). Cognitive behavioural therapy (CBT), the current gold-standard treatment, can reduce social anxiety in youth (Scaini et al. 2016) but many fail to show clinically significant responses (Kendall et al. 2012), respond but subsequently relapse (Ginsburg et al. 2014), or find it difficult to access. Identifying more effective, accessible methods so that young people can better manage their symptoms is a public health priority. Cognitive bias modification (CBM) training, which uses computerised tasks to target symptom-linked cognitive biases, has emerged as a potential adjunctive intervention (Butler et al. 2015; White et al. 2016) that may be amenable to delivery through computerised formats at home (Salemink et al. 2014) or in school (Fitzgerald et al. 2016). Yet, existing CBM packages remain weak at boosting more adaptive information-processing styles and at reducing symptoms (Cristea et al. 2015a, b; Heeren et al. 2015; Mogoaşe et al. 2014). This study presents a newly developed, multi-session computerised training program that targets multiple cognitive biases using a variety of training techniques and stimuli, for adolescents with elevated social fears. We assess the viability of administering this training tool at school, it’s acceptability to young people and compare changes in biases and symptoms across a baseline and an intervention phase.

Drawing on cognitive models of social anxiety (Clark and Wells 1995; Rapee and Heimberg 1997), a large corpus of research has found a link between social anxiety and attention and appraisal biases in adults as well as adolescents (Bar-Haim et al. 2007; Haller et al. 2016; Klein et al. 2017; Miers et al. 2008; Rheingold et al. 2003). These manifest as: greater allocation of attention to threatening stimuli at involuntary and voluntary stages of processing (Roy et al. 2008; Stirling et al. 2006); a tendency to interpret ambiguous cues in threatening ways; and a tendency to disproportionately attribute negative events as caused by oneself (i.e., ‘internal’ reasons) and positive events as caused by others or circumstance (i.e., ‘external’ reasons). Computerised cognitive training methods, which encourage more adaptive styles of information-processing over repeated trials and practice, have been developed in adults to reduce general and social anxiety. Cognitive bias modification of attention (CBM-A) methods alter maladaptive attention-orienting patterns towards threat, and encourage selective attention towards neutral or positive stimuli. Most commonly, CBM-A methods use a modified dot-probe task in which probes only ever appear in place of non-threatening stimuli (MacLeod et al. 1986). In contrast, in ‘visual search’ CBM-A training the individual must locate a non-threatening stimulus from among threatening stimuli as quickly as possible (Waters et al. 2013). Cognitive bias modification for interpretations (CBM-I) targets biases in interpretation, mostly using the ‘ambiguous situations task’ (Mathews and Mackintosh 2000). Here, participants read a series of ambiguous sentences that end with a word fragment. Completion of the final word disambiguates the valence of the sentence in a positive direction. Participants receive a follow-up ‘yes/no’ comprehension question with ‘correct/incorrect’ feedback in order to reinforce the training. A few studies have developed programs to modify attributions in adults to reduce depressive mood (Peters et al. 2011) but not anxiety.

However, studies of adults with various anxiety conditions (including trait anxiety) have only found weak (but significant effects) in symptom change (Hakamata et al. 2010; Hallion and Ruscio 2011; Heeren et al. 2015, but also see; Cristea et al. 2015a; Mogoaşe et al. 2014). Reduction in symptoms typically occur when there is also successful bias modification (MacLeod and Clarke 2015), and possibly through multiple training sessions (Hallion and Ruscio 2011). Extensions of CBM-A and CBM-I for use in adolescents (Bar-Haim et al. 2011; Lau et al. 2013), using the same tasks but with modifications to the stimuli content and modality (audio/text/pictures) have found small to medium effects of CBM-I and CBM-A training on cognitive biases, but no effect on general indices of mental health (nor on anxiety specifically) (Cristea et al. 2015b). Looking at these packages separately, Lowther and Newman (2014) identified that 8 out of 10 CBM-A studies reported positive changes in anxiety post-intervention (although only 4 of these 8 studies also found a change in attention bias). Through a meta-analysis, Krebs et al. (2017) found that CBM-I had a statistically significant moderate effect on decreasing negative interpretations and boosting positive interpretations. A small but significant effect on self-reported anxiety immediately following training was also found. While adult studies have tried to alter cognitive processes relating to depression through attribution training (Peters et al. 2011), their extension to young people has focused on targeting aggressive behaviours and academic achievements (Sukariyah and Assaad 2015; Vassilopoulos et al. 2015). No studies to our knowledge have trained adaptive attributions in adolescents (or adults) to reduce anxiety. Thus, while CBM training packages have potential, efforts to boost bias change and symptom reduction are needed. Adult data advocate multi-session training but their extension to anxious adolescents yield mixed findings regarding symptom and bias change for CBM-A (Fitzgerald et al. 2016; de Voogd et al. 2016, 2017b; Pergamin-Hight et al. 2016) and CBM-I (de Voogd et al. 2017a; Reuland and Teachman 2014). Therefore, consideration of other methodological factors may be important in prompting significant symptom change.

The current study aimed to improve CBM training effects by incorporating several methodological features into the training package. Some of these features drew directly on findings around known contributions of cognitive factors to anxiety, while others aimed to increase user-engagement. Consistent with combined cognitive bias hypotheses of psychopathology (Hirsch et al. 2006; Everaert et al. 2012, 2014), we first included bias modification procedures to target both attention and interpretation biases in social anxiety, within the same package. Targeting biases together may produce a greater magnitude of change (because of their combined additive and interactive effects). Only one study we are aware of has utilised a combined-bias approach in socially anxious adolescents (Sportel et al. 2013; de Hullu et al. 2017), testing an internet-based CBM-A/CBM-I program and finding significant improvement across all groups but no significant difference between internet-based CBM, CBT and control group. Secondly, CBM-A tasks aim to modify maladaptive processes of selective attention towards, and difficulty disengaging from, threatening environmental stimuli, yet do less to target self-focused attention. Models of social phobia (Clark and Wells 1995) posit that the socially-anxious individual shifts their attention inwards to produce an (often negative) image of themselves, based on interoceptive sources, rather than actual monitoring of others’ responses to disconfirm these negative fears. This self-focused attention in turn reduces processing of environmental cues in adults as well as adolescents (Hodson et al. 2008; Judah et al. 2016), which suggests that targeting these maladaptive self-focused attentional processes during CBM-A training could be beneficial (Wells and Papageorgiou 1998). We therefore included a task within the CBM-A package that draws the individual’s attention toward their internal feelings and then encourages them to shift their attention externally to stimuli that challenge these beliefs of how others view them in a social situation. Thirdly, we also increased the scope of CBM-I by targeting attribution biases too, particularly the tendency to internally attribute responsibility for negative events compared to positive events (Haller et al. 2016). We included a second task within the CBM-I package, that asked young people to generate an internal attribution for a positively interpreted event.

Finally, trial repetition, boredom and disengagement are serious concerns for CBM training (Beard 2011). We increased user-engagement by varying the training techniques used and the modality of stimulus presentation. A combination of CBM-A techniques was used, from the visual dot-probe to the visual search tasks. In the dot-probe, we trained attention towards positive words and faces on some blocks, and attention towards neutral words and faces on other blocks—always away from negative stimuli. In the visual search, participants identified a smiling face from a grid of negative faces in one module, but also practiced shifting their attention from internal sensations and cues toward benign, external interpersonal cues in another module. For CBM-I, we used text-based scenarios to encourage benign/positive resolution of ambiguous situations, as well as visual presentations of ambiguous scenes that had to be resolved benignly/positively. The latter may allow for more effective visualisation, and therefore stronger emotional responses and bias modification, than material presented in word form (Holmes et al. 2006; Holmes and Mathews 2010).

To assess viability and acceptability of our enhanced, multi-session CBM intervention for social anxiety, we used an A–B case series design, in which adolescents selected for high social anxiety received 8 school-based CBM training sessions, in two 4-day blocks over a 2-week period. We also gathered quantitative data on changes on selected measures during the 2-week intervention phase but also during a 2-week baseline period. We expected a significant decrease in social anxiety symptoms, and a significant change in attention and interpretation biases. Due to these clear a priori hypotheses, we conducted significance testing on changes in social anxiety symptoms, real-life socially avoidant behaviour and measures of attention and interpretation biases during the baseline versus the intervention phases. We also calculated the correlation between changes in social anxiety and changes in cognitive measures. To explore specificity effects to social anxiety symptoms, we measured changes on general anxiety and depression symptoms.

## Methods

### Design

**Fig. 1:**
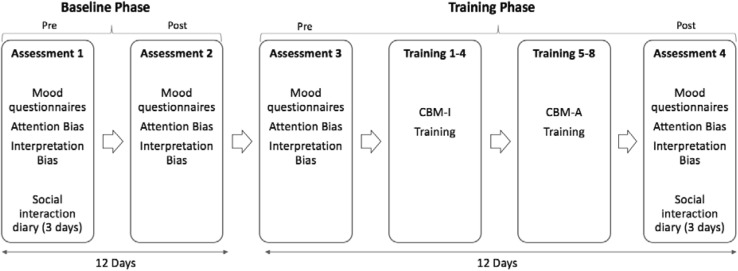
Schedule of assessment and training sessions for each participant

A single case series A–B design was used. Participants completed a 2-week baseline phase, followed by a 2-week intervention phase. Individual baselines acted as control periods to allow us to compare the effects of administrating the CBM programme on symptom and bias measures against any natural fluctuations over time. Self-reported measures of social anxiety, general anxiety, mood/depression, cognitive biases and responsiveness to real-life stressors were assessed before and after the 2-week multi-session CBM program, and also before and after the 2-week baseline phase, in which no training took place—resulting in 4 assessment time-points. As this study was carried out in secondary schools the procedure was designed to fit in with students’ schedules. Therefore, the pre-baseline phase assessment took place on the first Monday of the study, with the post-baseline phase assessment taking place on the Friday of the following week, 12 days later. After a 2-day break for the weekend, the pre-training phase assessment took place on the following Monday. Finally, the post-training phase assessment was carried out on the Friday of the following week, 12 days later. The use of 4 assessment time-points allowed for the comparison of pre-post changes over two distinct phases, one of which involved the CBM intervention. As the baseline and intervention phases were matched for duration, degree of change across pre/post assessment sessions could be directly compared within-subjects. See Fig. 1 for an illustration of the study timeline.

### Participants

Adolescents aged 16–18 years were recruited from two secondary schools in South London, England. Using an opt-out procedure, seventy-eight students (65 females and 13 males) completed the pre-screening Social Anxiety Scale for Adolescents (SAS-A). As teachers passed the information onto pupils and only those who were interested in taking part attended the screening session, it was difficult to calculate the initial recruitment rate and/or to assess the representativeness of those who did the screening. Using the recommended clinical cut-off of 50 (La Greca and Lopez 1998), 25 students (24 females) were invited to take part in the 4-week study. 22 females and 1 male agreed to participate but 4 of these dropped out prior to study completion, due to existing time commitments at school, leaving 19 participants who completed all training and assessment sessions (18 females and 1 male). Given that this study aimed to explore the preliminary effects associated with a multi-session, multi-bias enhanced CBM training program in adolescents, there were no prior studies upon which to base sample size calculations. Furthermore, the need for a priori power calculations for case series designs has been debated. Our final sample size was commensurate with the mean/median of other case series in the literature (Abeles et al. 2009; Bechor et al. 2014; Blackwell and Holmes 2010; Rozenman et al. 2011). We did not conduct a formal assessment of current and lifetime mental health diagnoses. Current mental health diagnoses were listed as an exclusion criterion in our information sheets and the participant was asked to confirm during the consenting procedure that they had no current or lifetime diagnoses and had never received treatment from a mental health service. As all participants were over 16 years, they provided informed consent. Ethical approval for this protocol was granted by the Psychiatry, Nursing and Midwifery Research Ethics Subcommittee, King’s College London (PNM/13/14-157). Sample characteristics and self-report scores on symptoms measures appear in Table 1.

**Table 1:** Sample characteristics and mean (standard deviation) of SAS-A, MFQ and SCARED at the four assessment points

	Baseline phase		Training phase	
Time-point	Pre	Post	Pre	Post
Session	Assessment 1	Assessment 2	Assessment 3	Assessment 4
N	19	19	19	19
Age (years)	17.03			
Social anxiety score (SAS-A)	63.68 (8.08)	63.05 (8.46)	62.74 (8.23)	58.32 (10.23)
MFQ—total score	29.53 (13.02)	32.63 (13.14)	29.84 (12.27)	22.47 (10.82)
SCARED—total score	42.68 (12.91)	43.53 (10.78)	40.58 (11.93)	35.74 (13.53)

*SAS-A* social anxiety scale for adolescents, *MFQ* mood and feelings questionnaire, *SCARED* screen for child anxiety related disorders

### Procedure

For an illustration of the study phases see Fig. 1. Both the 12-day baseline and intervention phase consisted of two (pre/post) assessment sessions, each lasting approximately 45 min but with the pre/post assessment sessions of the intervention phase either side of a block of eight CBM training sessions (each lasting around 15–20 min, and never longer than 30 min). Approximately 4-weeks after the initial screening, each participant was seen individually in a quiet classroom, supervised by a researcher, throughout each session of data collection, including the CBM training. During Assessment 1, on the first Monday of the baseline phase, participants completed questionnaires on social anxiety, general anxiety and depression symptoms, followed by cognitive bias measures. During this week participants were also asked to complete the Social interaction diary at the end of the day on Tuesday–Thursday, and email the responses to the researcher each evening. The questionnaires and cognitive bias measures were repeated for Assessment 2, the post-baseline assessment, on the Friday of the following week. On the following Monday, these measures were repeated again for Assessment 3, the pre-training assessment. Training sessions 1–4 were carried out on the Tuesday–Friday of the same week, and saw the participant complete one interpretation training task per day from the training program. The following Monday–Thursday consisted of training sessions 5–8, which saw the participant complete one attention training task per day from the training program. As the interpretation training was anticipated to be more engaging (based on adult findings—Beard et al. 2012), we hoped we would retain more participants by administering it first. During Assessment 4, on the Friday of the same week, participants completed the same battery of measures from the previous assessments to provide us with a post-training assessment. The week after the final session, participants were again asked to complete the social interaction diary at the end of the day on Tuesday–Thursday, and email the responses to the researcher each evening. At the end of the entire study each participant was provided with full debriefing.

### Materials

#### Enhanced CBM Training Intervention

##### CBM-I: Interpretation and Attribution Training

**Fig. 2:**
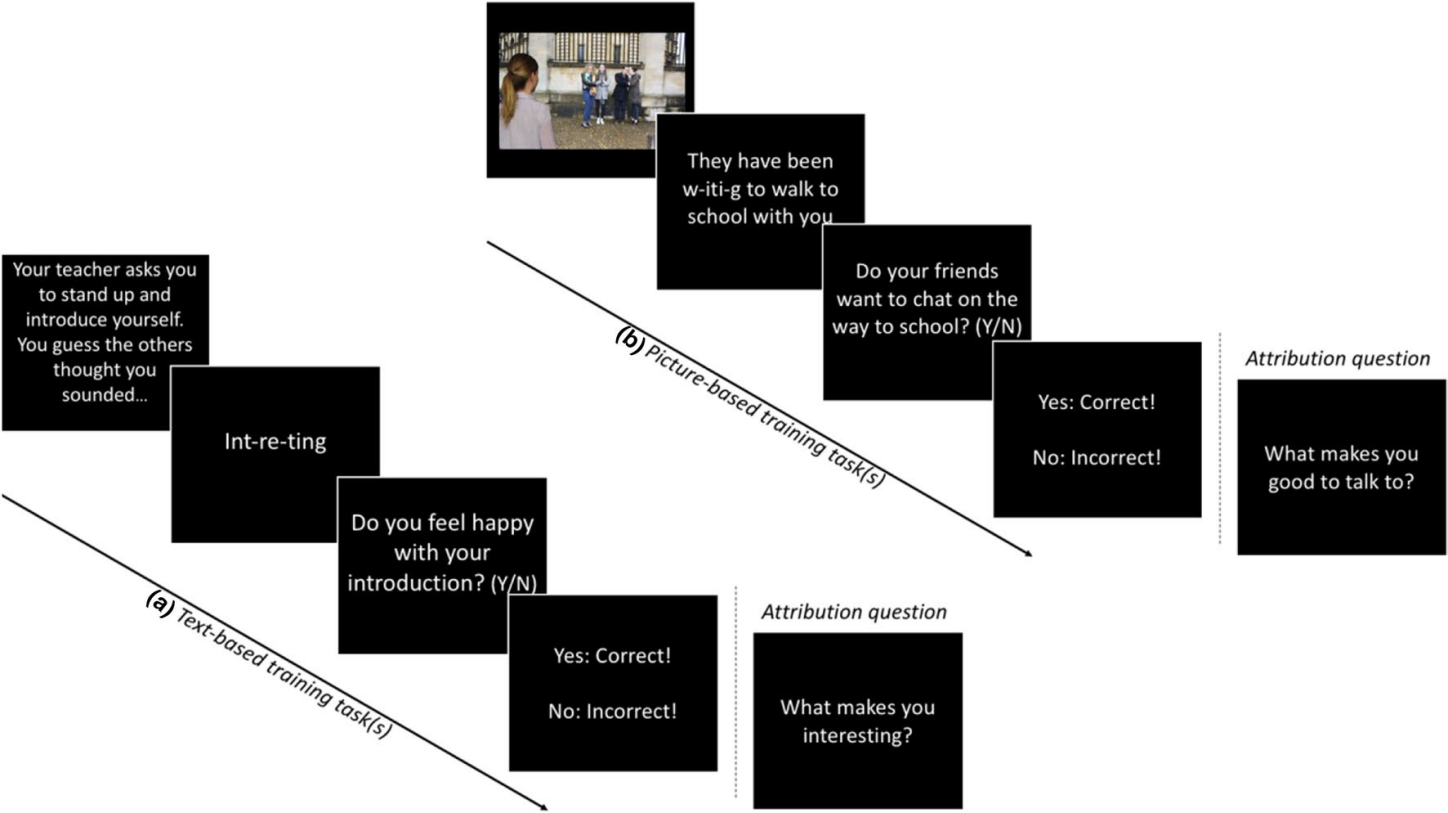
CBM-I training tasks: sequence *a* illustrates the text-based interpretation training tasks, with additional attribution question used in the attribution task variant. Sequence *b* illustrates the interpretation training tasks using picture scenes

This training segment consisted of 4 sessions—two of these used written vignettes to describe ambiguous social scenarios and two used picture scenes in an attempt to increase the vividness of ambiguous scenes (see Fig. 2). All picture stimuli used was from Haller et al. (2016, 2017) in which a new, picture-based tool was developed to measure interpretational and attributional biases of visual social cues in adolescents. The social situations used were based upon several previous adolescent CBM-I studies (Lau et al. 2013; Lothmann et al. 2011). During all interpretation training tasks participants were trained to endorse positive/benign rather than threatening interpretations in response to presentation of ambiguous, age-appropriate social scenarios. Session 1 presented participants with 25 text-based ambiguous situations that each ended with a word fragment in a positive or benign direction. Participants were asked to complete each fragment by typing in the correct letter. Correct completion disambiguated the scenario and a comprehension question followed, designed to reinforce the interpretation. For half of the comprehension questions, the correct answer was ‘yes’ and for the other ‘no’, so that they were not always positive. This was followed by a “correct/wrong” message. Session 2 was largely equivalent, but first used a picture scene to increase the vividness of the situation, which was then followed by a text-based description, with word fragment to complete, and comprehension question. As with the written descriptions in session 1, the initial picture scene presented to the participant in session 2 was always ambiguous. The text-based description with the word fragment after the picture was then designed to disambiguate the social scene in a benign or positive direction. Sessions 3 and 4 were identical to Sessions 1 and 2, but at the end of the interpretation component an additional question about attributions was posed to encourage participants to generate an internal attribution for the positively interpreted event. For instance, as outlined in Fig. 2, after training the participant to interpret an ambiguous event (approaching a group of friends waiting to chat with them) in a positive direction, they are then asked a question based upon this event (“What makes you good to talk to?”), encouraging them to attribute this positive outcome toward their own internal characteristics. This was an open response question in which the participant typed an answer using the keyboard. All sessions presented 25 interpretation trials.

##### CBM-A: Attention Training

**Fig. 3:**
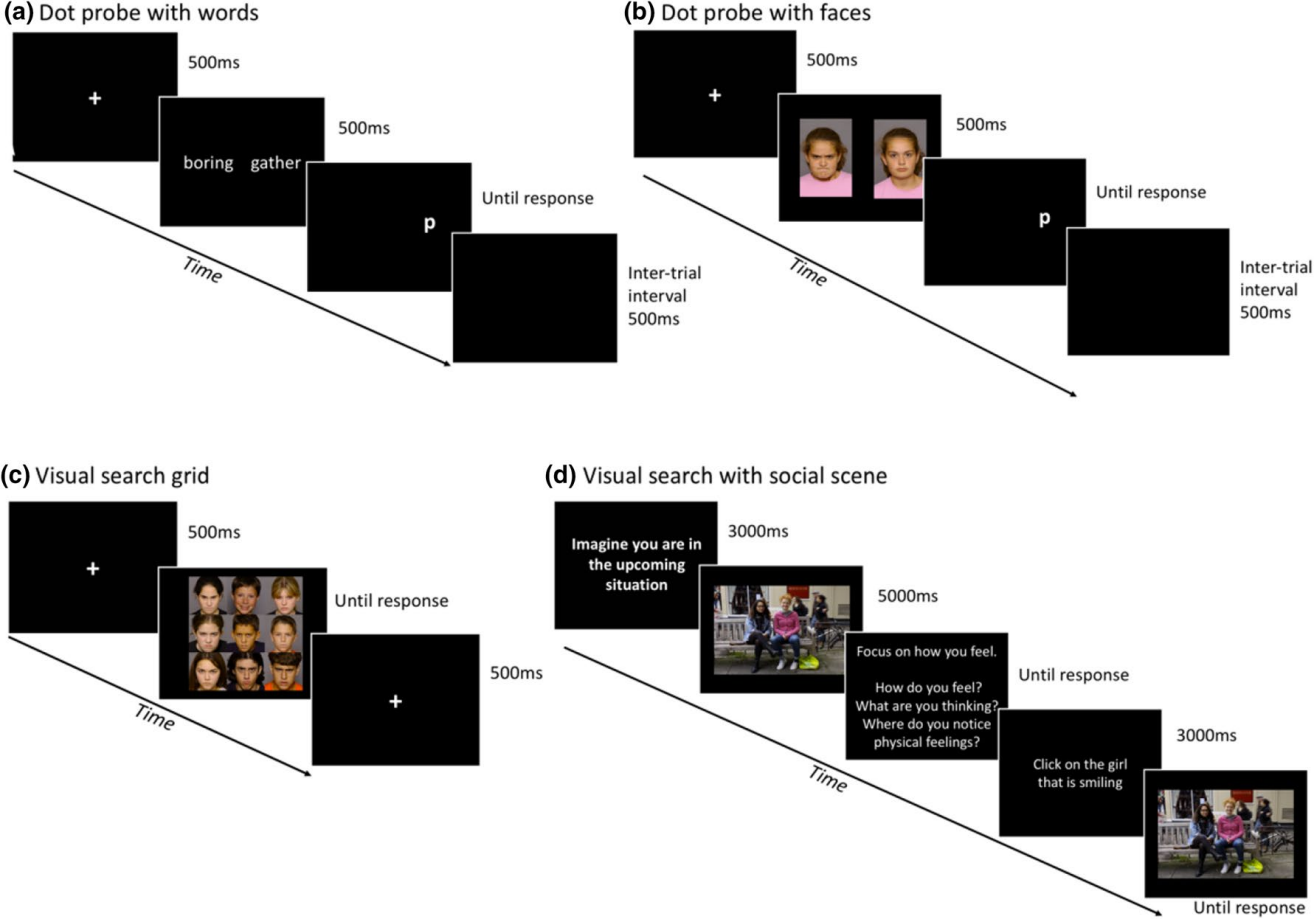
Sequence of stimuli presentation for each of the four attention training tasks

Of the four attention training sessions, two used the dot-probe task, and two used the visual search task. Figure 3 outlines each task.

*Dot-Probe Task* Of the dot-probe training sessions, one session used emotional faces while the second session used emotional words (see Fig. 3). We supplemented threat-neutral pairings with threat-positive pairings, to encourage attention towards positive stimuli, making it more commensurate with the visual search task. Emotional adolescent face stimuli (neutral, angry, happy) were used from the NIMH Child Emotional Faces Picture Set (NIMH-ChEFS; Egger et al. 2011). Participants viewed 160 trials (4 blocks of 40 trials) during each training session. Of these 64 were angry-neutral, 64 were angry-happy and 32 were neutral–neutral filler trials (interspersed to reduce chances of habituation to the expressions). Eight female and eight male faces were used. Each face pairing used the same actor. Each face pairing was shown four times for Angry–Happy and Angry–Neutral trials and twice for Neutral–Neutral trials. Each face photograph subtended 45 mm in width and 34 mm in height. The face photographs were presented with equal distance to the left and right of the fixation cross, with a distance of 14 mm between them. Each trial began with the presentation of a fixation display for 500 ms (white cross 1 × 1 cm at the centre of the screen), on which the participants were requested to focus their gaze. The fixation display was followed by a face pair display for 500 ms, immediately followed by a target probe (“p” or “q”); consistently in the location of the neutral or happy stimulus. Participants were required to locate the probe position and determine which symbol appeared by pressing one of the two pre-specified keys on the keyboard. The target remained on the screen until the participant responded. This meant that we were targeting attention biases at both voluntary and involuntary stages of processing, consistent with findings that anxiety symptoms have been associated with both (Lau and Waters 2017). An inter-trial-interval (500 ms) followed, before the next trial. A short break was given every 40 trials. Trials were presented in a randomised order. For session 2, the dot-probe word training task, participants viewed 160 trials; 64 Negative–Neutral, 64 Negative–Positive and 32 Neutral–Neutral socially relevant word pairings. 8 words were used 4 times each in the Negative–Neutral and Negative–Positive trials, and twice each the Neutral–Neutral Trials.

*Visual Search* This training paradigm again consisted of two sessions (see Fig. 3). Participants completed one session of visual search within a grid (based on Waters et al. 2013), in which they were required to repeatedly identify the only positive (smiling, mouth open) face in a 3 × 3 matrix of negative (angry, mouth closed) emotional faces. The faces used in this task were the same faces used in the dot-probe training. In the second session of visual search, they were presented with a relevant social scene, in which they were required to repeatedly identify a specified non-threatening face, along with questions designed to reduce self-focused attention and perspective taking to this external cue. The stimuli for this new task was also taken from Haller et al. (2016, 2017). We consciously chose stimuli that was not overly positive (relatively ambiguous), to attempt to mirror real-world situations the participant may encounter. After directing them to focus on their self-focused attention in response to this social-scene, we then prompted them to shift their attention externally to non-threatening stimuli in the social scene, that challenges their potentially negative beliefs of how others view them.

### Self-Report Symptom Measures

Questionnaire and diary measures assessed pre and post social anxiety symptoms and social interactions. The primary symptom measures were self-reported social anxiety and social interaction ratings. Measures of self-reported general anxiety and depression were collected to assess whether training effects were specific to social anxiety or had more general effects.

#### Social Anxiety

Social anxiety was measured using the Social Anxiety Scale for Adolescents (SAS-A; La Greca and Lopez 1998), a 22-item self-report measure of social anxiety symptoms. For the present study, internal consistency was α = 0.81 (using assessment 1 data), with test–retest reliability (using assessments 1 and 2 at baseline) at r = 0.86.

#### Social Interactions

This newly developed measure allowed participants to rate anxiety levels in response to real-life negative events using a self-report Visual Analogue Scale (VAS). Participants were asked how many negative interactions they experienced each day, to rate how “upset or angry” they felt immediately following their most negative interaction each day, from 0 (not at all) to 7 (extremely), and how “upset” or “angry” they subsequently felt (0–7). They were also asked to indicate how many potential social interactions they avoided each day. This questionnaire was provided as an email to the participant and treated as a “diary” to complete and return at the end of each day for 3 days (Tuesday–Thursday) during the first week of baseline phase, and 3 days (Tuesday–Thursday) the week following the training phase.

#### General Anxiety

Anxiety symptoms across dimensions of anxiety were measured using the Screen for Child Anxiety Related Disorders (SCARED; Birmaher et al. 1999), a 41 item self-report measure. For the present study, internal consistency was α = 0.89 (using assessment 1 data), with test–retest reliability at r = 0.91 (using assessments 1 and 2 at baseline).

#### Depression

Depression was measured using the Mood and Feelings questionnaire (MFQ; Costello and Angold 1988). For the present study, internal consistency was α = 0.92 (using assessment 1 data), with test–retest reliability at r = 0.83 (using assessments 1 and 2 at baseline).

### Cognitive Bias Measures

#### Interpretation Bias

##### Adolescent Interpretation Bias Task (AIBT; Heathcote et al. 2016)

This task consists of a series of incomplete vignettes describing ambiguous situations relevant to adolescent life. The task was originally created to investigate interpretation bias and the experience of pain in adolescents, and therefore consists of 8 vignettes relating to social situations and 8 relating to bodily threat. Only data from the social situations items were used for this study. After each vignette, the participants are presented with two different possible endings (negative or positive), which the they must rate in terms of whether or not that interpretation popped into their mind, on a scale of 1–5. Finally, participants are asked to select the interpretation that most readily popped into their mind. They then see all the situations again but this time must rate them based on their *belief* that each interpretation would actually be happening in that situation. Participants’ mean interpretation bias scores were calculated by subtracting total ratings of negative endings from total ratings of positive endings, when asked how likely the interpretation was to pop into their mind. A negative score indicates a bias toward negative interpretations. As our focus for this measure was on interpretation bias, we used the ‘likelihood’ rating scores as this was most commensurate with other measures of interpretation bias (Amin et al. 1998; Fu et al. 2013; Miers et al. 2008). However, changes on the ‘belief’ questions and on forced choice questions during the baseline and intervention phases were similar to those reported for the likelihood ratings, and are available from the first author on request. For the present study, test–retest reliability (using assessments 1 and 2 at baseline) was r = 0.88.

#### Attention Bias

##### Dot-Probe Task

The design of this task mirrored that used for the CBM-A training phase. This assessment task consisted of 160 trials: 80 trials of word stimuli, followed by 80 trials of face stimuli (32 neutral–angry trials, 32 neutral–happy trials and 16 neutral–neutral filler trials). The probe appeared with equal probability behind the emotional and neutral stimulus. Raw reaction time data for each participant was analysed (separately for words and faces) and trials with a response time ± 3 standard deviations for the participant’s mean were eliminated from further analyses (2.1% of all words trials, 1.8% of all faces trials). Trials with incorrect responses were also excluded (6.8% of all words trials, 6.5% of all faces trials). Participants who made incorrect or outlying responses on greater than 25% of trials were excluded from subsequent analyses. Following this, an attentional bias score was computed for each trial-type (Bias Score = ProbeNeutral − ProbeEmotion). Positive bias score values indicate a bias towards the emotion (vigilance bias) and negative values indicate a bias away from the emotion (avoidance bias). This was conducted separately for the dot-probe task using word stimuli and dot-probe task using face stimuli. Participants with an extreme bias score (± 3 standard deviations from the overall group mean) were excluded, resulting in the exclusion of one participant’s data from the dot-probe analyses. Based on our study aims, our results focus only on vigilance/avoidance of threat (i.e. Neutral–Threat trials). For the present study, test–retest reliability (using assessments 1 and 2 at baseline) was r = − 0.01.

### Feedback Questionnaire

At Assessment 4, participants were asked for their views of the program: “What were the most help/unhelpful aspects of the program?”; “Which parts of the program did you find the most enjoyable/unenjoyable?”; “Do you have any other comments on the program?”.

### Viability and Feedback

We assessed viability and participant acceptability by monitoring recruitment and drop-out rates. Responses to the feedback questions were collated into a database and salient themes were identified.

### Quantitative Data

Questionnaire total scores were calculated at four times points; pre- and post-baseline phase (Assessment 1 and 2), and pre- and post-training phase (Assessment 3 and 4) for each individual and presented in a Table. Due to a priori hypotheses, we performed statistical tests of the degree of change during the baseline versus intervention phase. These scores were entered into a 2 × 2 ANOVA with Phase (baseline and training) and Time (pre, and post) as the two within subject variables. Results of the self-report social interaction diary were collated; ratings of negative social interactions, immediate emotional response to the most negative interaction and number of social situations avoided over the days preceding the training program (during baseline) and the days following training (during the intervention) were again presented for all individuals. Paired samples t-tests were used to compare these pre- and post-assessment measures. Similarly, interpretation bias scores were presented for all 4 assessment time-points, and also entered into a 2 × 2 ANOVA with phase (baseline and training) and time (pre and post) as the two within subject variables. Attention bias scores for Neutral–Threat trials were also presented for each individual at all 4 assessment time-points and entered into a 2 × 2 ANOVA with phase and time as the two within subject variables. This was completed separately for words and faces conditions. A bivariate correlation analysis between change of attention bias, change of interpretation bias and change of symptoms (all Assessment 3—Assessment 4) is also presented. Bonferroni adjustment controlled for type 1 error in analyses where multiple ANOVAs were conducted, with adjusted p values reported.

## Results

### Descriptive Data and Baseline Associations

Mean scores for participants on questionnaire symptom measures of social anxiety, general anxiety and depression at each assessment are presented in Table 1. Table 2 reports correlations between cognitive bias measures with each other and with social anxiety symptom scores at baseline. None of these correlations reached significance.

**Table 2:** Correlations (r) between social anxiety scores on SAS-A and cognitive bias measures at baseline

Measure	AIBT	Dot-probe words	Dot-probe faces
SAS-A	− 0.356	− 0.381	− 0.316
*Significance (p)*	0.134	0.119	0.202
AIBT		0.385	0.093
*Significance (p)*		0.115	0.714
Dot-probe words			− 0.004
*Significance (p)*			0.986

Negative dot probe bias scores indicate an avoidance bias (toward neutral); negative AIBT bias scores indicate a proclivity toward negative interpretations

*SAS-A* social anxiety scale for adolescents, *AIBT* adolescent interpretation bias task (bias score), *Dot-probe words* dot probe bias score with word stimuli, *Dot-probe faces* dot probe bias score with face stimuli

### Viability and Feedback

19 the 23 participants completed the full CBM program—a retention rate of 82.6%.

Salient themes were identified from participant feedback responses: 67% of participants expressed that they found the social situations tasks generally felt helpful, with 45% claiming they thought the social situations task helped them in viewing situations more positively/less negatively. 33% of participants indicated they found the dot-probe tasks unhelpful, with the remaining 67% consisting of mainly “N/A” responses. A full list of feedback responses can be found in Table 3.

**Table 3:** Qualitative feedback from participants regarding their experience of the CBM training program

Participant	Feedback
1	Felt it helped realise that not every situation should be interpreted negatively. Dot-probe task didn’t feel helpful or enjoyable. Liked having to imagine themselves in certain situations, and try to think positively about them
2	Felt that being given a “correct answer” for the visual scenarios kind of made them rethink their interpretation of the situation
3	Felt they were able to think more positively about situations after training
4	Enjoyed the situations task and found it helpful seeing them in a different more positive angle after a while. Preferred the descriptions to pictures. At some points it felt too long, especially on dot-probe task. It was pretty easy to understand and follow and helped them think about situations more positively
5	They felt the interpretation bias tasks with pictures were helpful. In fact, the whole “situations” part of the programme felt helpful. They didn’t feel like the dot probe tasks felt like they were helping in any way
6	Thought it was all “ok”
7	Didn’t feel like it was helpful
8	Felt the part where they had to look at how others perceived them was helpful. The dot probe task felt pointless. They liked the fact it was computerised and there wasn’t too much one on one talking. The dot-probe tasks were quite confusing
9	They felt it possibly allowed them to view social situations in a more positive way. The sessions were long. The tasks themselves were very boring and repetitive. They felt the programme itself does not help to reduce the way they view social situations but now the aim has been more thoroughly explained they may begin to view their own social interactions differently, more positively. Felt the word-based scenarios allowed them to better imagine themselves in the situation than the pictures
10	Didn’t enjoy answering the open questions
11	The social situations task felt helpful
12	The questionnaires made them question why they have been stressing so much. They feel they have become much more calm, especially coming to school, because they would usually be nervous about the day in general before coming in. Didn’t see the point in the dot probe task
13	Felt it was helpful when imagining different scenarios to see how they would react to them. Enjoyed the detecting the smiles game (visual search). Felt the scenarios were too repetitive
14	Felt imagining themselves in situations was helpful. The dot probe task didn’t feel beneficial
15	Thought the tasks should be shorter
16	Felt the social scenario tasks were helpful. Thought the picture-based scenarios were less helpful than the word-based ones
17	Thought it was beneficial to realistically look at how certain scenarios won’t play out as badly as they think
18	They found the social scenario questions useful in relating them to the reality of decision making. They found the tasks very simple and straightforward to understand
19	It helped them realise that not every situation should be interpreted negatively. The dot probe task didn’t feel helpful or enjoyable. Enjoyed imagining themselves in certain situations, and trying to think positively about them. Felt some of the tasks lasted too long

Participants were asked “Were there any aspects of the program that you found particularly helpful/unhelpful?”, “Were there any aspects of the program that you particularly liked/disliked?” The responses above are a collation of these answers

### Quantitative Data

#### Quantitative Data: Individual Questionnaire Scores

Each participants’ scores on the social anxiety, general anxiety and depression symptom measures at the 4 assessment time-points are displayed in Table 4. Individual participants’ scores on the social interaction diary items are displayed in Table 5.

**Table 4:** Sample characteristics and total SAS-A, MFQ and SCARED scores for each participant at Assessment 1 (pre-baseline phase), Assessment 2 (post-baseline phase), Assessment 3 (pre-training phase) and Assessment 4 (post training phase)

Participant	Age	Sex	Baseline phase						Training phase					
			Assessment 1 (pre)			Assessment 2 (post)			Assessment 3 (pre)			Assessment 4 (post)		
			SAS-A	SCARED	MFQ	SAS-A	SCARED	MFQ	SAS-A	SCARED	MFQ	SAS-A	SCARED	MFQ
1^a^	18.00	F	47	32	27	49	35	21	47	23	16	43	20	21
2	17.25	F	62	42	28	63	38	32	60	30	36	64	31	38
3	16.58	F	60	37	18	60	36	23	60	34	22	64	38	23
4	16.75	F	63	33	18	56	37	24	51	38	15	57	35	13
5^a^	16.83	M	63	52	30	68	51	28	66	52	31	59	49	39
6	16.58	F	74	61	43	76	57	49	69	55	34	75	49	37
7	16.67	F	83	56	27	84	53	32	83	51	28	85	51	39
8^a^	16.75	F	59	39	24	62	44	39	60	34	35	54	37	23
9^a^	17.33	F	68	43	15	63	48	17	63	35	13	46	29	2
10^a^	17.25	F	63	25	15	60	29	15	60	24	19	59	27	14
11^a^	17.92	F	58	64	45	56	65	38	63	66	46	56	54	25
12^a^	16.33	F	66	44	37	67	49	48	66	41	16	62	30	19
13	17.08	F	53	17	13	59	34	31	58	29	37	58	15	10
14^a^	16.75	F	59	35	22	58	39	29	58	39	37	51	32	21
15^a^	16.92	F	58	36	33	56	31	30	61	37	34	52	15	20
16^a^	16.92	F	75	49	59	75	44	60	77	43	60	68	47	31
17^a^	17.00	F	68	31	24	56	27	12	56	31	18	48	18	6
18^a^	17.92	F	65	58	29	69	59	39	67	57	29	59	60	23
19^a^	16.75	F	66	57	54	61	51	53	67	52	41	48	42	23

*SAS-A* social anxiety scale for adolescents, *MFQ* mood and feelings questionnaire, *SCARED* screen for child anxiety related disorders

^a^Individuals for whom social anxiety scores showing a reduction from assessment 3–4

**Table 5:** Mean scores on the social interaction diary items, for each participant pre and post training.

Participant	Pre-training			Post-training		
	Negative interactions	Situations avoided	Emotional response	Negative interactions	Situations avoided	Emotional response
1	0.5	0.5	3	1	1	5
2^a^	3	2	3	0.5	1.5	1.5
3	0.5	0	2	0.5	0	2
4	0.5	0	2	0.5	0	2
5	0	0.5	0	0.5	0.5	7
6	4	1.5	4.5	5	3.5	6.5
7^a^	2.5	5.5	4.5	1.5	3.5	4.5
8^a^	1	0.5	4	0.5	0	5
9	0	0	0	0	0	0
10^a^	3.5	4.5	2	1.5	1	2
11	–	–	–	–	–	–
12	1	4.5	3	1	0.5	3
13	1	0.5	4	0.5	2	5
14^a^	1.5	1.5	4	1	0	2
15^a^	1	3.5	5.5	0.5	0.5	3
16^a^	1.5	2	5.5	0.5	1	2
17^a^	2	1.5	3.5	1	1.5	1
18^a^	1	2	6	0.5	0.5	5
19^a^	1.5	2	2	0.5	0.5	3

Negative interactions = mean scores of the number of negative social interaction reported via the social interaction diary over a 3-day period during the baseline phase (pre-training) and after the training phase (post-training). Situations avoided = mean scores of number of potentially negative social situations avoided (again via self-report diary) over the same 3-day periods, pre and post training. Emotional response = mean scores of how upset or angry the participant felt immediately after their most negative interaction each day, on a scale of 0–7. Participant 11 had email issues and therefore was unable to receive/send any questionnaires

^a^Individuals for whom at least two of the social interaction diary items showed a reduction from assessment 3–4

#### Quantitative Data: Changes in Social Anxiety

Across participants, significant main effects of phase [F(1,18) = 11.68, p = .003, η_p_^2^ = 0.39] and time [F(1,18) = 5.70, p = .028, η_p_^2^ = 0.24] and their interaction [F(1,18) = 5.14, p = .036, η_p_^2^ = 0.22] emerged. Decomposing the interaction showed that the extent to which social anxiety scores differed across time varied with phase. Tests of simple main effects showed that SAS-A means were not significantly different between pre-baseline and pre-treatment assessments but instead decreased significantly between the post-baseline and post-training assessments [F(1,18) = 14.27, p = .001, η_p_^2^ = 0.44]. Moreover, SAS-A means were not significantly different from pre-baseline to post-baseline, but did significantly decrease pre-training to post-training [F(1,18) = 7.49, p = .014, η_p_^2^ = 0.29]. Looking at the individual data, particularly social anxiety symptom scores at assessments 3 and 4 for, suggested some variability in symptom improvement; 68% of the 19 participants showed a reduction in symptoms across session assessments 3 and 4; although it is worth noting that the range in reported symptom reduction was large (− 1 to − 19; individuals who reported a reduction are marked with * in Table 4). A minority (26%) of participants showed an increase in symptoms (+ 2 to + 6) and one participant reported no change.

#### Quantitative Data: Changes in Social Interactions

Across participants, a significant reduction in the number of negative social interactions experienced from pre (M = 2.89, SD = 2.30) to post (M = 1.89, SD = 2.17) training [t(17) = 2.47, p = .024, *d* = 0.58] was found. The number of social interactions avoided also significantly reduced from pre (M = 3.61, SD = 3.36) to post (M = 1.94, SD = 2.18) training [t(17) = 2.21, p = .041, *d* = 0.52]. A paired samples t-test on affect ratings showed a significant reduction in immediate emotional response pre (M = 6.63, SD = 3.26) to post (M = 4.38, SD = 2.92) training [t(15) = 2.45, p = .02, *d* = 0.61]. Data for individual participants showed that only 53% of the 18 participants with valid data showed a reduction on at least two of the items from the social diary assessments from pre to post-training.

#### Quantitative Data: Changes in General Anxiety

There was a significant main effect of phase [F(1,18) = 24.77, p < .001, η_p_^2^ = 0.58] as well as a significant time-by-phase interaction [F(1,18) = 6.73, p = .018, η_p_^2^ = 0.27]. Post hoc analyses revealed that SCARED means were not significantly different pre-baseline and pre-treatment assessments, but did significantly decrease from post-baseline and post-training assessments [F(1,18) = 22.19, p < .001, η_p_^2^ = 0.55]. SCARED means were not significantly different pre-baseline and post-baseline assessments, however did significantly decrease from pre-training to post-training assessments [F(1,18) = 8.21, p = .01, η_p_^2^ = 0.31].

#### Quantitative Data: Changes in Depression

The main effect of phase [F(1,18) = 6.05, p = .024, η_p_^2^ = 0.25] and the interaction between time and phase [*F*(1,18) = 10.51, p = .*005*, η_p_^2^ = 0.37] were significant. Tests of simple main effects for phase and time found that MFQ means were significantly decreased between post-baseline and post-training assessments [F(1,18) = 13.36, p < .002, η_p_^2^ = 0.43], but not between pre-baseline and pre-training assessments. For effects of time, there was a significant reduction between pre- and post-training assessments, [F(1,18) = 7.54, p = .013, η_p_^2^ = 0.30], but not between pre- and post-baseline assessments.

#### Quantitative Data: Changes in Cognitive biases

Participant mean scores on the assessments of interpretation and attention biases are presented in Table 6.

**Table 6:** Mean (standard deviation) of AIBT and dot probe scores

	Baseline phase		Training phase	
Time-point	Pre	Post	Pre	Post
Session	Assessment 1	Assessment 2	Assessment 3	Assessment 4
N	19	19	19	19
AIBT bias score	− 6.37(11.16)	− 3.95 (10.57)	− 4.21 (9.72)	1.32 (9.28)
AIBT—total positive ratings	13.16 (5.58)	16.42 (5.50)	16.95 (6.03)	19.42 (5.87)
AIBT—total negative ratings	19.53 (6.03)	20.37 (5.61)	21.16 (4.94)	18.11 (5.84)
Dot probe bias score—words (ms)*	− 18.1 (113.12)	44.3 (79)	26.3 (63.39)	5.4 (66.72)
RT—neutral	678.6 (181.24)	614.2 (122.22)	591.4 (123.88)	525.5 (87.89)
RT—threat	696.7 (259.73)	569.9 (77.74)	565.1 (98.14)	520.1 (71.1)
Dot probe bias score—faces (ms)*	11.9 (67.4)	21.7 (81.6)	22.1 (53.9)	15.2 (42.0)
RT—neutral	634.1 (125.2)	558.5 (59.0)	567.0 (109.3)	514.0 (74.9)
RT—threat	622.2 (112.6)	580.3 (117.1)	589.1 (104.6)	498.7 (60.3)

*AIBT* adolescent interpretation bias task, *RT* reaction time, *ms* milliseconds

*N = 18 for dot probe data due to exclusion of outlier

#### Interpretation Bias

Main effects for both phase [F(1,18) = 7.08, p = .016, η_p_^2^ = 0.28] and time [F(1,18) = 18.29, p < .001, η_p_^2^ = 0.50] were statistically significant but not their interaction. Nonetheless, given the observed large decrease in bias score pre- to post-training, and given that our a priori predictions were that changes would happen during training phase, a post-hoc one-way ANOVA was run to explore main effects of time during each phase. There was no significant main effect of time during the baseline phase [F(1,18) = 3.75, p = .069, η_p_^2^ = 0.44], however there was a significant increase in bias score (more positive than negative interpretations) between pre- and post-training assessments [F(1,18) = 14.25, p = .001, η_p_^2^ = 0.44]. Furthermore, there was significant increase between post-baseline and post-training assessments [F(1,18) = 7.79, p = .012, η_p_^2^ = 0.30], and no significant difference between pre-baseline and pre-training assessments.

#### Attention Bias

Analysis of the neutral–threat (faces) dot-probe data found no significant effects. The same analysis of the neutral–threat dot-probe using words found neither of the main effects for phase or time reached statistical significance, however the interaction between time and phase was significant [F(1,17) = 6.07, p = .025, η_p_^2^ = 0.26]. Post hoc analyses found that bias score means significantly differed only between post-baseline and post-training assessments [F(1,17) = 4.8, p = .043, η_p_^2^ = 0.22]. No significant effects were found for neutral–positive dot-probe bias scores using words. The same analysis of the neutral–positive dot-probe using faces found main effects for both phase [F(1,17) = 5.90, p = .026, η_p_^2^ = 0.26] and time [F(1,17) = 5.30, p < .034, η_p_^2^ = 0.24] were statistically significant but not their interaction.

#### Quantitative Data: Correlations Between Change in Symptoms and Change in Biases

Bivariate correlations showed that increased interpretation bias scores on the AIBT from pre- to post-training (i.e. an increased readiness to interpret ambiguous events less negatively) was significantly associated with reductions in SAS-A scores pre- to post-training (r = − 0.56, p = .012). Correlations between change in attention bias scores, as measured by Dot-Probe bias scores for selective attention toward threat (words), and change in symptom scores on the SAS-A, pre- to post-training, were not significant. There was also no significant correlation between change in symptom scores on the SAS-A and change in selective attention bias scores toward threat when using face stimuli.

## Discussion

This case series explored the value of a combined-bias, multi-session CBM program, for adolescents with elevated social anxiety. While targeting both attention and interpretation biases for threat, new training modules targeting self-focused attention and internal attributions were included as well as a variety of training techniques that used both verbal and pictorial stimuli to enhance user-engagement. The data obtained suggest that it is viable to deliver this CBM program in a school in individual sessions with a trained researcher. Under experimental conditions, the program showed itself to be feasible in terms of its applicability and accessibility in a school setting: Only 4 of the 23 participants withdrew from the study prior to completion, thus it appears to have a good acceptability from participants. Although not directly assessed, school teachers were largely supportive of this research and we had good recruitment rates amongst schools. It should be noted that participants were always accompanied by a researcher and some participants received several reminders of their appointment and needed supervision by a researcher in order to remain engaged in the training task. Some participants were fully engaged throughout the entire study without additional support from the researcher. This has implications for determining whether a CBM program such as the one used in this study, delivered in a school, is engaging enough for individuals to complete without supervision.

The significant reduction in symptoms on the SAS-A following eight sessions of CBM over 2-weeks, compared to no significant reduction in SAS-A scores following a 2-week baseline phase—and similar findings using a diary measure of daily social interactions—suggests that there is some potential in reducing social anxiety levels in adolescents reporting elevated symptoms. However, there are two caveats to this conclusion. First, although 13 participants showed changes on social anxiety symptoms, these varied between a decrease of 1–19, across the training phase, possibly suggesting that a few individuals with large changes drove the significant decreases. Also, only around 9 showed reductions across items on the social diary assessment. This suggests variability in how useful this training was for targeting social anxiety across individuals, reflected somewhat in the qualitative feedback too. Second, data from other outcome measures showed that these effects were not specific to social anxiety, and instead reductions in depressive and general anxiety symptoms were also observed. It is possible the observed decrease in socially-avoidant behaviour led to increased exposure to potentially rewarding social situations, thus having an impact on these general affective indices. However, as all of these measures were self-report, we cannot rule out the possibility that these broader symptoms changes indicate the presence of demand effects.

Also challenging for our findings of symptom improvement was mixed findings around changes in interpretation and attention bias. Although post-hoc analysis showed that interpretation bias scores did show a significant change pre- to post-training with no significant change pre to post baseline, the absence of a significant interaction effect between phase and time suggests that the degree of change was not significantly greater. However, individual scores show that for most participants the interpretation bias went in the intended direction, and several participants showed a greater jump from Assessment 3 to Assessment 4 than from Assessment 1 to Assessment 2. The feedback, on the whole, also points to several participants feeling the CBM-I tasks were beneficial. Finally, there was a significant association between this change in interpretative style and change in social anxiety symptoms. It should be noted that, whilst there was a lack of significant correlation between initial baseline interpretation bias and SAS, the correlation reported was in the expected direction. With a larger sample size, we would expect this to reach significance. Furthermore, a weak correlation between initial interpretation bias and SAS may not be a prerequisite of a correlation between changes in these two variables, if the common factor explaining this correlation is the administration of a training tool designed to effect changes on both. Therefore, we tentatively suggest biased interpretations could provide a promising target for symptom improvement for some young people.

In contrast, we found no significant effects for attention bias change, or any correlation between change in attention bias to threat and change in symptoms. There was also a lack of significant correlation between cognitive biases and symptom measures at baseline. It may be that our current method for assessing attention bias is problematic. Previous research has shown the dot-probe task has poor reliability, comprising internal consistency and test–retest reliability in children and adolescents (Brown et al. 2014; White et al. 2016) and in adults (Van Bockstaele et al. 2017). Indeed, the current results display an extremely low test–retest reliability for attention bias r = − 0.01, compared to the interpretation bias measure (r = 0.88). Some studies using eye-tracking have demonstrated that certain measures, such as dwell time across trials on socially threatening stimuli, are more reliable across time, but also more consistent in their associations with social anxiety (Lazarov et al. 2017). More generally, others have argued that a visual search grid could be more effective than the dot probe as a tool for more reliably measuring and more effectively modifying attention processes that are linked to anxiety (Mogg and Bradley 2016; Van Bockstaele et al. 2017). The development and application of potentially more stable and reliable measures like these are essential to better understand the nature and modification of attentional biases. Furthermore, as participant feedback suggests that the rigidity of the dot-probe task may result in a lack of motivation and task engagement, incorporating extrinsic motivators, such as real-time performance feedback (Bernstein and Zvielli 2014) and using this real-time performance data to tailor the task to the individual’s optimal rate of learning (Schnyer et al. 2015), may increase task engagement and improve attention bias change.

While the training task was generally acceptable, the feedback collected provides more insights into further features that could improve effectiveness and engagement. Participant feedback suggests that as the goal of the CBM-I training portion became clearer, it gradually gave the participant an understanding of not needing to view social situations so negatively. It may be that incorporating explicit instructions to practice the target bias may enhance CBM efficiency (MacLeod et al. 2009). This could be particularly true for the CBM-A tasks, as feedback suggests participants found these tasks ‘boring’ and ‘un-engaging’, partly due to not understanding why they were doing them. Feedback regarding task-specific elements of the CBM program suggests that, contrary to expectations, word-based social situations were in fact more successful in creating visual imagery than the picture based scenarios. Several participants found the unfamiliar visual stimuli harder to engage and immerse than the word-based descriptions. Use of more personalised picture stimuli may be of greater use perhaps with incorporation of media such as videos to improve immersion for the participant. This feedback is in line with recent research finding no difference in outcome when attempting to improve CBM-I effectiveness by incorporating visual imagery (de Voogd et al. 2017a). Whilst we have no way of quantitatively assessing whether this study benefited from multiple versus single sessions of training, the qualitative feedback suggests that after several sessions of CBM-I training some participants experienced increased insight, that they could ‘look at social situations less negatively’. This may point towards a combination of implicit and explicit processes—with implicit training effects on processing, extending to influence ongoing behaviour via increased insight. Finally, participant feedback does show some variation in specific aspects of the program that participants found helpful/engaging, which may partially explain individual differences found in symptom reduction.

Whilst the symptom changes on social anxiety are encouraging, the data also provide several challenges. That this study was a preliminary case series with a small sample size, the appropriateness of significance-testing of statistical comparisons is questionable with different approaches taken in prior studies, (Abeles et al. 2009; Bechor et al. 2014; Blackwell and Holmes 2010; Rozenman et al. 2011). However, we limited our statistical tests to key measures that related to a priori expectations. Second, although there are advantages to carrying out a A–B case series in the same participants (self-matching means that any potential confounders such as socioeconomic status, genetic risk, state of health etc., are automatically controlled for), the absence of an active control group or condition means we are unable to attribute symptom change directly to cognitive training procedures (over a placebo effect). Furthermore, this program was presented to participants as a new psychological training program designed to target cognitive biases, which may have increased demand effects and expectancy biases [see MacLoed et al. (2009 ) for a more thorough discussion of this issue]. Use of questions to reveal expectancy beliefs (Schmidt et al. 2009) may be beneficial for future studies in assessing the possibility of demand effects. Additionally, as all participants completed the baseline phase prior to training and our design did not include a control group, we are unable to fully account for natural fluctuations in anxiety across time. However, as a first-step, such case series is important as performing a cross-over case series design and a randomised controlled clinical trial may be premature, and not an optimal strategy for investing research and patient resources. Third, the lack of bias effects might be to do with mixed training, as none of the training tasks were completed for more than two sessions. Fourth, the design could have benefited from a follow-up time point, with the possibility that all consequences of CBM may take a longer time to become evident. Previous CBM research has found that emotional outcomes continue post-CBM completion (Schmidt et al. 2009). Finally, the generalisability of our findings was affected by the strong gender disparity in our sample: Female pupils self-selecting into such studies have been a feature of school-based recruitment in many of our studies. As students were allowed to ‘opt out’ of the screening procedure, there was little we could do to change this. Despite these limitations, we find the study has provided some encouraging findings. The CBM program has demonstrated its potential as an easily accessible resource for adolescents with elevated social anxiety. The next step will be to test these tasks in a larger sample with a comparison condition or group.

## References

[CR1] Abeles P, Verduyn C, Robinson A, Smith P, Yule W, Proudfoot J (2009). Computerized CBT for adolescent depression (“Stressbusters”) and its initial evaluation through an extended case series. Behavioural and Cognitive Psychotherapy.

[CR2] Amin N, Foa EB, Coles ME (1998). Negative interpretation bias in social phobia. Behaviour Research and Therapy.

[CR3] Bar-Haim Y, Lamy D, Pergamin L, Bakermans-Kranenburg MJ, Van Ijzendoorn MH (2007). Threat-related attentional bias in anxious and nonanxious individuals: A meta-analytic study. Psychological Bulletin.

[CR4] Bar-Haim Y, Morag I, Glickman S (2011). Training anxious children to disengage attention from threat: A randomized controlled trial. Journal of Child Psychology and Psychiatry.

[CR5] Beard C (2011). Cognitive bias modification for anxiety: Current evidence and future directions. Expert Review of Neurotherapeutics.

[CR6] Beard C, Weisberg RB, Primack J (2012). Socially anxious primary care patients’ attitudes toward cognitive bias modification (CBM): A qualitative study. Behavioural and Cognitive Psychotherapy.

[CR7] Bechor M, Pettit JW, Silverman WK, Bar-Haim Y, Abend R, Pine DS, Jaccard J (2014). Attention bias modification treatment for children with anxiety disorders who do not respond to cognitive behavioral therapy: A case series. Journal of Anxiety Disorders.

[CR8] Bernstein A, Zvielli A (2014). Attention feedback awareness and control training (A-FACT): Experimental test of a novel intervention paradigm targeting attentional bias. Behaviour Research and Therapy.

[CR9] Birmaher B, Brent DA, Chiappetta L, Bridge J, Monga S, Baugher M (1999). Psychometric properties of the Screen for Child Anxiety Related Emotional Disorders (SCARED): A replication study. Journal of the American Academy of Child & Adolescent Psychiatry.

[CR10] Blackwell SE, Holmes EA (2010). Modifying interpretation and imagination in clinical depression: A single case series using cognitive bias modification. Applied Cognitive Psychology.

[CR11] Brown HM, Eley TC, Broeren S, MacLeod C, Rinck MHJA, Hadwin JA, Lester KJ (2014). Psychometric properties of reaction time based experimental paradigms measuring anxiety-related information-processing biases in children. Journal of Anxiety Disorders.

[CR12] Butler E, Mobini S, Rapee RM, Mackintosh B, Reynolds SA (2015). Enhanced effects of combined cognitive bias modification and computerised cognitive behaviour therapy on social anxiety. Cogent Psychology.

[CR13] Clark DM, Wells A (1995). A cognitive model of social phobia. Social Phobia: Diagnosis, Assessment, and Treatment.

[CR14] Costello EJ, Angold A (1988). Scales to assess child and adolescent depression: Checklists, screens, and nets. Journal of the American Academy of Child & Adolescent Psychiatry.

[CR15] Cristea IA, Kok RN, Cuijpers P (2015). Efficacy of cognitive bias modification interventions in anxiety and depression: Meta-analysis. The British Journal of Psychiatry.

[CR16] Cristea IA, Mogoașe C, David D, Cuijpers P (2015). Practitioner review: Cognitive bias modification for mental health problems in children and adolescents: A meta-analysis. Journal of Child Psychology and Psychiatry.

[CR17] de Hullu E, Sportel BE, Nauta MH, de Jong PJ (2017). Cognitive bias modification and CBT as early interventions for adolescent social and test anxiety: Two-year follow-up of a randomized controlled trial. Journal of Behavior Therapy and Experimental Psychiatry.

[CR18] de Voogd EL, de Hullu E, Heyes SB, Blackwell SE, Wiers RW, Salemink E (2017). Imagine the bright side of life: A randomized controlled trial of two types of interpretation bias modification procedure targeting adolescent anxiety and depression. PloS ONE.

[CR19] de Voogd EL, Wiers RW, Prins PJM, De Jong PJ, Boendermaker WJ, Zwitser RJ, Salemink E (2016). Online attentional bias modification training targeting anxiety and depression in unselected adolescents: Short-and long-term effects of a randomized controlled trial. Behaviour Research and Therapy.

[CR20] de Voogd EL, Wiers RW, Salemink E (2017). Online visual search attentional bias modification for adolescents with heightened anxiety and depressive symptoms: A randomized controlled trial. Behaviour Research and Therapy.

[CR21] Egger HL, Pine DS, Nelson E, Leibenluft E, Ernst M, Towbin KE, Angold A (2011). The NIMH Child Emotional Faces Picture Set (NIMH-ChEFS): A new set of children’s facial emotion stimuli. International Journal of Methods in Psychiatric Research.

[CR22] Everaert J, Duyck W, Koster EH (2014). Attention, interpretation, and memory biases in subclinical depression: A proof-of-principle test of the combined cognitive biases hypothesis. Emotion.

[CR23] Everaert J, Koster EH, Derakshan N (2012). The combined cognitive bias hypothesis in depression. Clinical Psychology Review.

[CR24] Fitzgerald A, Rawdon C, Dooley B (2016). A randomized controlled trial of attention bias modification training for socially anxious adolescents. Behaviour Research and Therapy.

[CR25] Fu X, Du Y, Au S, Lau JY (2013). Reducing negative interpretations in adolescents with anxiety disorders: A preliminary study investigating the effects of a single session of cognitive bias modification training. Developmental Cognitive Neuroscience.

[CR26] Ginsburg GS, Becker EM, Keeton CP, Sakolsky D, Piacentini J, Albano AM, Peris T (2014). Naturalistic follow-up of youths treated for pediatric anxiety disorders. JAMA Psychiatry.

[CR27] Hakamata Y, Lissek S, Bar-Haim Y, Britton JC, Fox NA, Leibenluft E, Pine DS (2010). Attention bias modification treatment: A meta-analysis toward the establishment of novel treatment for anxiety. Biological Psychiatry.

[CR28] Haller SP, Doherty BR, Duta M, Kadosh KC, Lau JY, Scerif G (2017). Attention allocation and social worries predict interpretations of peer-related social cues in adolescents. Developmental Cognitive Neuroscience.

[CR29] Haller SP, Raeder SM, Scerif G, Kadosh KC, Lau JY (2016). Measuring online interpretations and attributions of social situations: Links with adolescent social anxiety. Journal of Behavior Therapy and Experimental Psychiatry.

[CR30] Hallion LS, Ruscio AM (2011). A meta-analysis of the effect of cognitive bias modification on anxiety and depression. Psychological Bulletin.

[CR31] Heathcote LC, Koopmans M, Eccleston C, Fox E, Jacobs K, Wilkinson N, Lau JY (2016). Negative interpretation bias and the experience of pain in adolescents. The Journal of Pain.

[CR32] Heeren A, Mogoașe C, Philippot P, McNally RJ (2015). Attention bias modification for social anxiety: A systematic review and meta-analysis. Clinical Psychology Review.

[CR34] Hirsch CR, Clark DM, Mathews A (2006). Imagery and interpretations in social phobia: Support for the combined cognitive biases hypothesis. Behavior Therapy.

[CR35] Hodson KJ, McManus FV, Clark DM, Doll H (2008). Can Clark and Wells‘(1995) cognitive model of social phobia be applied to young people?. Behavioural and Cognitive Psychotherapy.

[CR36] Holmes EA, Mathews A (2010). Mental imagery in emotion and emotional disorders. Clinical Psychology Review.

[CR37] Holmes EA, Mathews A, Dalgleish T, Mackintosh B (2006). Positive interpretation training: Effects of mental imagery versus verbal training on positive mood. Behavior Therapy.

[CR38] Judah MR, Grant DM, Carlisle NB (2016). The effects of self-focus on attentional biases in social anxiety: An ERP study. Cognitive, Affective, & Behavioral Neuroscience.

[CR39] Kendall PC, Settipani CA, Cummings CM (2012). No need to worry: The promising future of child anxiety research. Journal of Clinical Child & Adolescent Psychology.

[CR40] Klein AM, de Voogd L, Wiers RW, Salemink E (2017). Biases in attention and interpretation in adolescents with varying levels of anxiety and depression. Cognition and Emotion.

[CR41] Krebs G, Pile V, Grant S, Degli Esposti M, Montgomery P, Lau JY (2017). Research review: Cognitive bias modification of interpretations in youth and its effect on anxiety: A meta-analysis. Journal of Child Psychology and Psychiatry.

[CR42] La Greca AM, Lopez N (1998). Social anxiety among adolescents: Linkages with peer relations and friendships. Journal of Abnormal Child Psychology.

[CR43] Lau JY, Belli SR, Chopra RB (2013). Cognitive bias modification training in adolescents reduces anxiety to a psychological challenge. Clinical Child Psychology and Psychiatry.

[CR45] Lau JY, Waters AM (2017). Annual research review: An expanded account of information-processing mechanisms in risk for child and adolescent anxiety and depression. Journal of Child Psychology and Psychiatry.

[CR47] Lazarov A, Pine DS, Bar-Haim Y (2017). Gaze-contingent music reward therapy for social anxiety disorder: A randomized controlled trial. American Journal of Psychiatry.

[CR48] Lothmann C, Holmes EA, Chan SW, Lau JY (2011). Cognitive bias modification training in adolescents: Effects on interpretation biases and mood. Journal of Child Psychology and Psychiatry.

[CR49] Lowther H, Newman E (2014). Attention bias modification (ABM) as a treatment for child and adolescent anxiety: A systematic review. Journal of Affective Disorders.

[CR50] MacLeod C, Clarke PJ (2015). The attentional bias modification approach to anxiety intervention. Clinical Psychological Science.

[CR51] MacLeod C, Koster EH, Fox E (2009). Whither cognitive bias modification research? Commentary on the special section articles. Journal of Abnormal Psychology.

[CR52] MacLeod C, Mathews A, Tata P (1986). Attentional bias in emotional disorders. Journal of Abnormal Psychology.

[CR53] Mathews A, Mackintosh B (2000). Induced emotional interpretation bias and anxiety. Journal of Abnormal Psychology.

[CR55] Miers AC, Blöte AW, Bögels SM, Westenberg PM (2008). Interpretation bias and social anxiety in adolescents. Journal of Anxiety Disorders.

[CR56] Mogg K, Bradley BP (2016). Anxiety and attention to threat: Cognitive mechanisms and treatment with attention bias modification. Behaviour Research and Therapy.

[CR57] Mogoaşe C, David D, Koster EH (2014). Clinical efficacy of attentional bias modification procedures: An updated meta-analysis. Journal of Clinical Psychology.

[CR59] Owens M, Stevenson J, Norgate R, Hadwin JA (2008). Processing efficiency theory in children: Working memory as a mediator between trait anxiety and academic performance. Anxiety, Stress, & Coping.

[CR60] Pergamin-Hight L, Pine DS, Fox NA, Haim B (2016). Attention bias modification for youth with social anxiety disorder. Journal of Child Psychology and Psychiatry.

[CR61] Peters KD, Constans JI, Mathews A (2011). Experimental modification of attribution processes. Journal of Abnormal Psychology.

[CR62] Rapee RM, Heimberg RG (1997). A cognitive-behavioral model of anxiety in social phobia. Behaviour Research and Therapy.

[CR63] Reuland MM, Teachman BA (2014). Interpretation bias modification for youth and their parents: A novel treatment for early adolescent social anxiety. Journal of Anxiety Disorders.

[CR64] Rheingold AA, Herbert JD, Franklin ME (2003). Cognitive bias in adolescents with social anxiety disorder. Cognitive Therapy and Research.

[CR65] Roy AK, Vasa RA, Bruck M, Mogg K, Bradley BP, Sweeney M, CAMS Team (2008). Attention bias toward threat in pediatric anxiety disorders. Journal of the American Academy of Child & Adolescent Psychiatry.

[CR66] Rozenman M, Weersing VR, Amir N (2011). A case series of attention modification in clinically anxious youths. Behaviour Research and Therapy.

[CR67] Salemink E, Kindt M, Rienties H, Van Den Hout M (2014). Internet-based cognitive bias modification of interpretations in patients with anxiety disorders: A randomised controlled trial. Journal of Behavior Therapy and Experimental Psychiatry.

[CR69] Scaini S, Belotti R, Ogliari A, Battaglia M (2016). A comprehensive meta-analysis of cognitive-behavioral interventions for social anxiety disorder in children and adolescents. Journal of Anxiety Disorders.

[CR70] Schmidt NB, Richey JA, Buckner JD, Timpano KR (2009). Attention training for generalized social anxiety disorder. Journal of Abnormal Psychology.

[CR71] Schnyer DM, Beevers CG, Sherman SM, Cohen JD, Norman KA, Turk-Browne NB (2015). Neurocognitive therapeutics: From concept to application in the treatment of negative attention bias. Biology of Mood & Anxiety Disorders.

[CR72] Sportel BE, de Hullu E, de Jong PJ, Nauta MH (2013). Cognitive bias modification versus CBT in reducing adolescent social anxiety: A randomized controlled trial. PLoS ONE.

[CR73] Stirling LJ, Eley TC, Clark DM (2006). Preliminary evidence for an association between social anxiety symptoms and avoidance of negative faces in school-age children. Journal of Clinical Child and Adolescent Psychology.

[CR74] Sukariyah MB, Assaad G (2015). The effect of attribution retraining on the academic achievement of high school students in mathematics. Procedia-Social and Behavioral Sciences.

[CR75] Van Bockstaele B, Salemink E, Bögels SM, Wiers RW (2017). Limited generalisation of changes in attentional bias following attentional bias modification with the visual probe task. Cognition and Emotion.

[CR76] Vassilopoulos SP, Brouzos A, Andreou E (2015). A multi-session attribution modification program for children with aggressive behaviour: Changes in attributions, emotional reaction estimates, and self-reported aggression. Behavioural and Cognitive Psychotherapy.

[CR77] Waters AM, Pittaway M, Mogg K, Bradley BP, Pine DS (2013). Attention training towards positive stimuli in clinically anxious children. Developmental Cognitive Neuroscience.

[CR78] Wells A, Papageorgiou C (1998). Social phobia: Effects of external attention on anxiety, negative beliefs, and perspective taking. Behavior Therapy.

[CR79] White LK, Britton JC, Sequeira S, Ronkin EG, Chen G, Bar-Haim Y, Pine DS (2016). Behavioral and neural stability of attention bias to threat in healthy adolescents. Neuroimage.

[CR81] Wittchen HU, Stein MB, Kessler RC (1999). Social fears and social phobia in a community sample of adolescents and young adults: Prevalence, risk factors and co-morbidity. Psychological Medicine.

[CR82] Woodward LJ, Fergusson DM (2001). Life course outcomes of young people with anxiety disorders in adolescence. Journal of the American Academy of Child & Adolescent Psychiatry.

